# Hypoaminoacidemia underpins glucagon-mediated energy expenditure and weight loss

**DOI:** 10.1016/j.xcrm.2022.100810

**Published:** 2022-11-15

**Authors:** David C.D. Hope, Charlotte E. Hinds, Tatiana Lopes, Matthew L. Vincent, Jed V. Shrewsbury, Arthur T.C. Yu, Iona Davies, Rebecca Scott, Ben Jones, Kevin G. Murphy, James S. Minnion, Alessandro Sardini, David Carling, Thomas A. Lutz, Stephen R. Bloom, Tricia M.M. Tan, Bryn M. Owen

**Affiliations:** 1 Division of Diabetes, Endocrinology and Metabolism, Department of Metabolism, Digestion and Reproduction, Imperial College London, London, UK; 2 MRC London Institute of Medical Sciences, Imperial College London, London, UK; 3 Institute of Veterinary Physiology, Vetsuisse Faculty, University of Zurich, Zurich, Switzerland

**Keywords:** glucagon, obesity, weight loss, energy expenditure, low protein, amino acids

## Abstract

Glucagon analogs show promise as components of next-generation, multi-target, anti-obesity therapeutics. The biology of chronic glucagon treatment, in particular, its ability to induce energy expenditure and weight loss, remains poorly understood. Using a long-acting glucagon analog, G108, we demonstrate that glucagon-mediated body weight loss is intrinsically linked to the hypoaminoacidemia associated with its known amino acid catabolic action. Mechanistic studies reveal an energy-consuming response to low plasma amino acids in G108-treated mice, prevented by dietary amino acid supplementation and mimicked by a rationally designed low amino acid diet. Therefore, low plasma amino acids are a pre-requisite for G108-mediated energy expenditure and weight loss. However, preventing hypoaminoacidemia with additional dietary protein does not affect the ability of G108 to improve glycemia or hepatic steatosis in obese mice. These studies provide a mechanism for glucagon-mediated weight loss and confirm the hepatic glucagon receptor as an attractive molecular target for metabolic disease therapeutics.

## Highlights

• G108, a long-acting glucagon analog, stimulates hepatic amino acid catabolism
• Energy expenditure and weight loss is a response to G108-stimulated hypoaminoacidemia
• Dietary protein supplementation rescues plasma amino acids
• G108 improves glycemia and hepatic steatosis independent of dietary protein

## Graphical abstract

**Figure:**
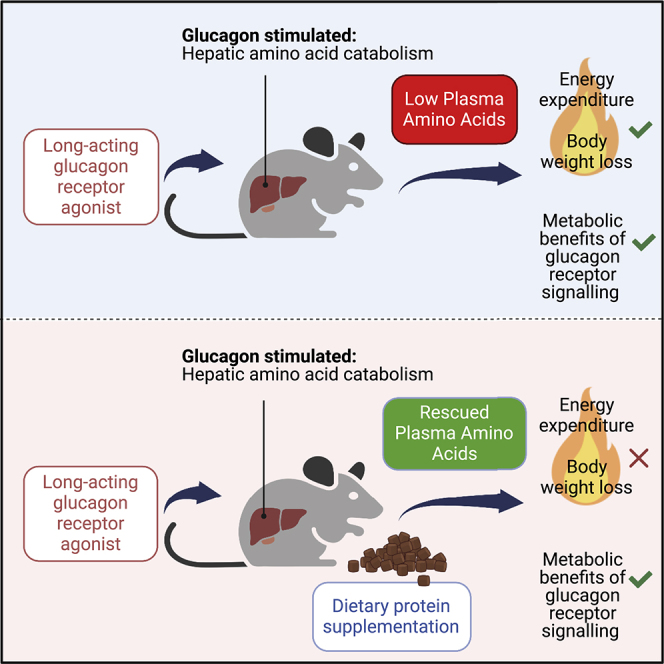

## Teaser

Using a rationally designed GCGR agonist, G108, Hope et al. demonstrate that glucagon-mediated energy expenditure is driven by a whole-body response to low plasma amino acid availability. However, neither energy expenditure nor weight loss are required for improvements in fatty liver disease or blood glucose control in obese mice.

## Introduction

Glucagon, a 29-amino acid peptide hormone secreted from the pancreatic alpha-cell, stimulates hepatic glucose production and maintains blood glucose concentrations during fasting.^1^ Glucagon also plays an important role in the hepatic catabolism of lipids and amino acids,^2^ pancreatic beta-cell insulin secretion,^3^ regulation of food intake,^4^^,^^5^ and energy expenditure.^6^

Given its effect on hepatic glucose mobilization, it is counterintuitive that glucagon could be used as a component of anti-obesity pharmacotherapies for individuals with, or at risk of, type 2 diabetes. Indeed, glucagon antagonism has previously been mooted as a therapeutic strategy.^7^ However, when combined with glucagon-like peptide-1 (GLP-1) in humans, glucagon has been shown to enhance energy expenditure (EE) and reduce food intake, with preservation of glycemic neutrality in response to meals.^8^^,^^9^ Several multi-agonists with GLP-1 receptor (GLP-1R) and glucagon receptor (GCGR) activity are currently being developed to treat metabolic disease.^10^

Pre-clinical studies have confirmed that GCGR activation contributes to the metabolic benefits of GLP-1R/GCGR co-agonism by increasing hepatic lipid catabolism and driving weight loss by enhancing EE.^11^^,^^12^^,^^13^^,^^14^ The effects on lipid catabolism appear to be a result of direct signaling at the level of the hepatocyte.^15^ However, the mechanisms driving EE are not fully understood. Early *in vitro* studies implicated brown adipose tissue (BAT) in glucagon-mediated thermogenesis in rodents; however, GCGR signaling within BAT does not increase whole-body EE in response to exogenous glucagon *in vivo.*^16^^,^^17^ Furthermore, acute infusion studies in humans have also shown that glucagon-induced EE is not mediated by BAT activation,^18^ suggesting that there are BAT-independent mechanisms for this phenomenon.

Hepatic GCGR activation upregulates pathways of hepatic amino acid catabolism and reduces plasma amino acids.^19^^,^^20^^,^^21^ These findings are potentially important for two reasons. First, therapeutic use of GCGR-targeted multi-agonists could result in hypoaminoacidemia-induced loss of lean mass. Second, observations from models of dietary protein or amino acid restriction^22^^,^^23^^,^^24^^,^^25^ raise the prospect that glucagon-mediated EE may be a response to low plasma amino acid availability. Both possibilities currently remain unexplored. However, their relevance is highlighted by recent clinical studies reporting hypoaminoacidemia associated with GCGR-targeted multi-agonists.^26^^,^^27^^,^ It is therefore essential to determine the chronic effect of GCGR agonism on plasma amino acids and how this influences lean mass, EE, and body weight. It is also important to determine the effect of strategies to mitigate hypoaminoacidemia, for example increased dietary protein intake. Together, these findings may have implications for anti-obesity therapeutics targeting the GCGR.

To determine the effects of chronic glucagon-stimulated hypoaminoacidemia on lean mass, EE, and body weight, we have developed a long-acting GCGR agonist, G108, which possesses similar activities to native glucagon. We established the anorectic threshold of G108 and showed that at doses below this, it caused hypoaminoacidemia and functional muscle loss in lean mice. We also show that dietary protein supplementation in obese mice rescues plasma amino acids, protects against lean mass loss, and eliminates G108-mediated EE and total body weight loss. Consistent with models of dietary protein restriction,^22^^,^^24^^,^^28^ mechanistic studies confirmed that depletion of plasma amino acids is a pre-requisite for G108-mediated EE and body weight loss. Despite these findings, our studies also confirm that glucagon is an attractive metabolic disease therapeutic as it improves glycemia and hepatic steatosis in the context of dietary protein supplementation, an intervention that helps to prevent lean mass loss.

## Results and discussion

### A long-acting glucagon-receptor agonist, G108, potently stimulates hepatic carbohydrate and amino acid metabolic pathways *in vivo*

**Figure 1:**
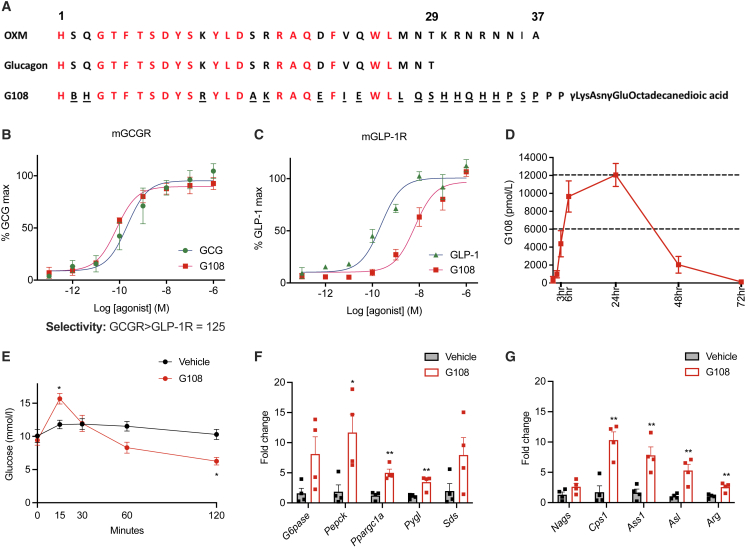
A long-acting glucagon-receptor agonist, G108, potently stimulates hepatic carbohydrate and amino acid metabolic pathways *in vivo* (A) Amino acid sequence of oxyntomodulin, glucagon, and G108. Red letters indicate conserved amino acids, and underlined letters modified amino acids. B, aminoisobutyric acid. (B) Stimulation of cAMP accumulation by glucagon (GCG) and G108 via mouse GCGR in HEK293T cells. n = 4. (C) Stimulation of cAMP accumulation by GLP-1 and G108 via mouse GLP-1R in HEK293T cells. n = 4. (D) Pharmacokinetic profile in rats given 0.5 mg G108. n = 4. (E) Non-fasted venous blood glucose at baseline and up to 2 h following a single dose of G108 (10 nmol/kg) in lean mice. n = 6/group. Significance indicates change compared with vehicle group. (F and G) Hepatic gene expression in (F) the gluconeogenesis pathway and (G) the urea cycle 24 h following a single dose of G108 (20 nmol/kg) in lean mice. n = 4/group. *G6pase*, glucose 6-phosphatase; *Pepck*, phosphoenolpyruvate carboxykinase; *Ppargc1a*, peroxisome proliferator-activated receptor gamma coactivator 1-alpha; *Pygl*, glycogen phosphorylase; *Sds*, serine dehydratase; *Nags*, N-acetylglutamate synthetase; *Cps1*, carbamoyl phosphate synthetase 1; *Ass1*, arginosuccinate synthetase 1; *Asl*, arginosuccinate lyase; *Arg*, arginase. ∗p < 0.05, ∗∗p < 0.01, data plotted as mean ± SEM. Gene expression data analyzed by the 2^−ΔΔCT^ method, normalized to cyclophilin endogenous control, unpaired t test. Glucose profile analyzed using two-way ANOVA and post hoc Sidak test.

We have generated a library of peptide analogs of oxyntomodulin and glucagon with varying receptor specificities, as well as lipidated C termini to increase their circulating half-lives through reversible binding to serum albumin. One such compound, G108, is a highly potent and selective GCGR agonist, with over a 120-fold selectivity factor for the GCGR over the GLP-1R (Figures 1A–1C; Table S1), which is comparable to native glucagon.^3^

Pharmacokinetic analysis demonstrated the long-acting nature of G108 following a single dose, with peak plasma concentrations achieved at 24 h (Figure 1D). As expected, a single dose of G108 in mice caused an acute increase in blood glucose (Figure 1E). Furthermore, we found that G108 increased the expression of hepatic gluconeogenic enzymes at a time point that coincided with peak plasma concentrations of the analog (Figure 1F). In keeping with the known effects of glucagon in rodents,^19^ G108 also increased the expression of a number of genes involved in amino acid catabolism (Figure 1G). As such, G108 represents a long-acting GCGR agonist that, *in vivo*, potently stimulates hepatic carbohydrate and protein catabolic pathways.

### G108 causes hypoaminoacidemia and lean mass loss via the hepatic GCGR

Previous studies, including from our own laboratory, have demonstrated a loss of lean mass in mice with GLP-1R/GCGR co-agonists^29^^,^^30^ and a highly selective GCGR agonist.^31^ Due to the amino acid catabolic properties of glucagon,^20^ and the importance of plasma amino acid availability for lean mass maintenance,^32^ we reasoned that these findings may be a result of glucagon-stimulated hypoaminoacidemia. We therefore initially used G108 as a long-acting glucagon analog to explore this important question.

**Figure 2:**
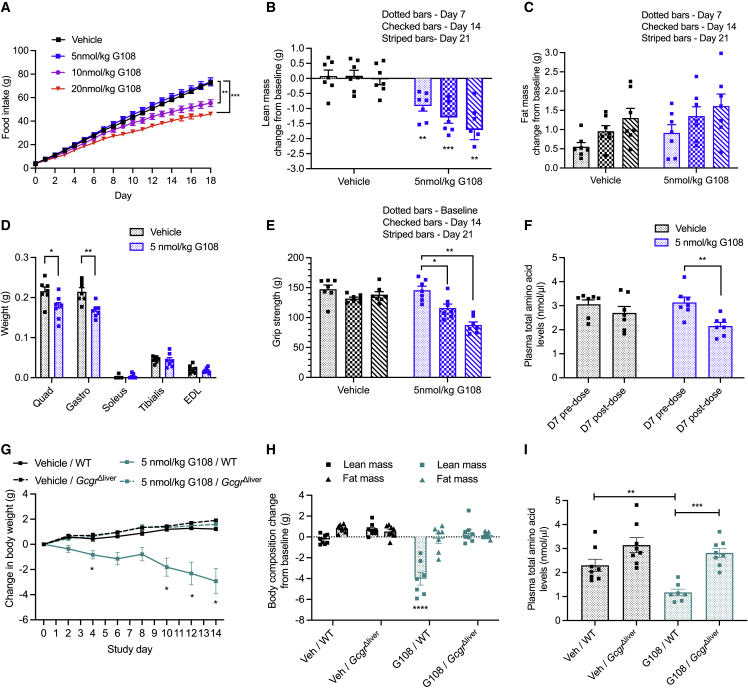
G108 causes hypoaminoacidemia and lean mass loss via the hepatic glucagon receptor (A) Food intake during an 18-day dose finding study with daily administration of vehicle (black) or 5 (blue), 10 (purple), or 20 (red) nmol/kg G108 in lean mice. n = 6/group. (B and C) Change in (B) lean and (C) fat mass from baseline over 21 days with daily dosing of 5 nmol/kg G108 or vehicle control in lean mice. Dotted = day 7, checked = day 14, striped = day 21. n = 7/group. Significance indicates change compared with vehicle control. (D) Muscle weights (g) on day 21 of study. n = 7/group. (E) Forelimb grip strength (g). Dotted = baseline, checked = day 14, striped = day 21. n = 7/group. (F) Plasma total amino acids on day 7 of study. n = 7/group. (G) Change in body weight in wild-type (WT) and *Gcgr*^Δliver^ mice following 14 days of daily administration with 5 nmol/kg G108. Significance indicates change from baseline. n = 7–8/group. (H) Change in body composition in WT and *Gcgr*^Δliver^ mice after 14 days of daily G108 (5 nmol/kg) administration. n = 7–8/group. Significance indicates change compared with vehicle (Veh)/WT group. (I) Plasma total amino acids in WT and *Gcgr*^Δliver^ mice after 14 days of daily administration with 5 nmol/kg G108. n = 7–8/group. ∗p < 0.05, ∗∗p < 0.01, ∗∗∗p < 0.001, ∗∗∗∗p < 0.0001, data plotted as mean ± SEM. Analyzed using a combination of t test and one- and two-way ANOVA with post hoc Sidak and Dunnett tests.

We first aimed to establish the anorectic threshold of G108 in lean mice. We found that doses of 10 and 20 nmol/kg G108 caused a sustained reduction in food intake (Figure 2A). However, daily injections of G108 at 5 nmol/kg had no effect on food intake (Figure 2A) but prevented weight gain compared with vehicle over 21 days (Figures S1A and S1B). Body composition analysis revealed that at this sub-anorectic dose, the suppression of weight gain was almost exclusively a result of reduced lean mass (Figures 2B and 2C). The weight of the quadriceps and gastrocnemius muscles (Figure 2D) and grip strength (Figure 2E) were all reduced in G108-treated animals, indicating functional muscle loss. G108 also caused a sustained reduction in plasma amino acids (Figure 2F), indicative of increased amino acid catabolism and explaining the effects on muscle mass. These effects were mediated by hepatic GCGR signaling as mice lacking the hepatic GCGR were completely refractory to the effects of G108 on body weight, lean mass, and plasma amino acids (Figures 2G–2I).

Together, these findings demonstrate that the long-acting glucagon analog G108 potently stimulates hypoaminoacidemia via hepatic GCGR signaling and that this is associated with a reduction in muscle function in lean mice. As hypoaminoacidemia has been recently associated with GCGR-targeted agonists in clinical studies,^26^^,^^27^ our findings suggest that strategies to prevent hypoaminoacidemia and preserve lean mass may be important for their long-term use in a therapeutic setting.

### Dietary protein supplementation prevents G108-mediated EE and body weight loss in obese rodents

As GCGR agonism has been proposed as a weight loss strategy,^33^ we aimed to determine whether dietary protein supplementation would preserve lean mass in obese animals treated with G108. Based on previous studies implicating hypoaminoacidemia in the EE response to dietary protein or amino acid restriction,^22^^,^^23^^,^^24^^,^^25^ we also set out to establish whether rescuing low plasma amino acids would influence G108-mediated EE and body weight loss in obese animals. For these studies in diet-induced obese (DIO) mice, we slightly increased the dose of G108 to 7.5 nmol/kg to counteract any obesity-associated hepatic glucagon resistance^21^ and found that this dose still did not affect food intake (Figure S2A).

**Figure 3:**
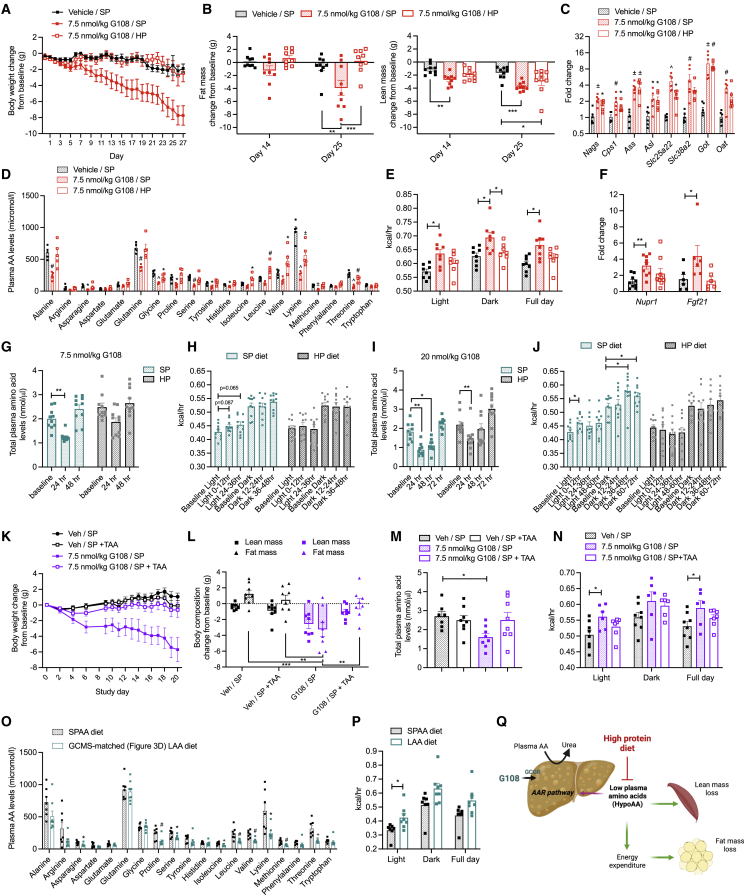
Dietary protein supplementation prevents G108-mediated EE and body weight loss through rescue of hypoaminoacidemia in obese mice (A) Change in body weight over 27 days in DIO mice treated daily with 7.5 nmol/kg G108. Standard protein (SP) diet = filled bars/symbols, high protein (HP) diet = open bars/symbols (Table S2). n = 9/group. (B) Change in fat and lean mass (g) from baseline after 14 and 25 days of daily dosing with G108. (C) Expression of markers of hepatic amino acid catabolism, N-acetylglutamate synthetase (*Nags*), carbamoyl phosphate synthetase 1 (*Cps1*), arginosuccinate synthetase 1 (*Ass1*), arginosuccinate lyase (*Asl*), solute carrier family 25 member 22 (*Slc25a22*), solute carrier family 38 member 2 (*Slc38a2*), glutamic oxaloacetic transaminase 1 (*Got1*), and ornithine aminotransferase (*Oat*). n = 5–6/group. Significance denotes change compared with Veh control group. (D) Individual plasma amino acids after 13 days of daily dosing with G108. n = 5/group. Significance denotes change compared with Veh control group. (E) Average energy expenditure (kcal/h). n = 7–8/group. (F) Fold change in expression of nuclear protein-1 (*Nupr1*) and fibroblast growth factor-21 (*Fgf21*). n = 6–9/group. (G) Plasma total amino acids in mice administered with a single dose of 7.5 nmol/kg G108, provided with an SP or HP diet (Table S2). n = 10/group. (H) Average energy expenditure (kcal/h) up to 48 h following a single dose of 7.5 nmol/kg G108. n = 10/group. (I) Plasma total amino acids in mice administered with a single dose of 20 nmol/kg G108, provided with SP or HP diet. n = 9–10/group. (J) Average energy expenditure (kcal/h) up to 72 h following single dose of 20 nmol/kg G108. n = 9–10/group. Significance denotes individual time point relative to photoperiod baseline. (K) Change in body weight over 20 days in DIO mice treated daily with 7.5 nmol/kg G108, provided with either SP diet (Table S2) or an SP diet supplemented with individual total amino acids (SP + TAA: formula 1; Table S3). n = 8/group. (L) Change in lean and fat mass (g) from baseline after 18 days of daily dosing with G108. n = 8/group. (M) Plasma total amino acids after 16 days of daily dosing with G108. n = 7–8/group. (N) Average energy expenditure (kcal/h). n = 6–8/group. (O) Individual plasma amino acids in DIO mice after 34 days of receiving either an amino acid-based SP diet (SPAA diet: formula 4; Table S3) or a low amino acid diet (LAA diet: formula 5; Table S3), matched to G108-stimulated reduction in individual plasma amino acids as shown in (D). n = 7–8/group. (P) Average energy expenditure (kcal/h) in mice receiving either SPAA or LAA diet. n = 7–8/group. (Q) Schematic highlighting effects of G108-stimulated hypoaminoacidemia. ∗p < 0.05, # or ∗∗p < 0.01, ± or ∗∗∗p < 0.001, ^ or ∗∗∗∗p < 0.0001. Data plotted as mean ± SEM. Gene expression data analyzed by the 2^−ΔΔCT^ method and normalized to cyclophilin endogenous control. EE data analyzed using regression based ANCOVA using lean mass or total mass as a covariate. Body weight data analyzed using repeated measures linear mixed model. All other data analyzed using a combination of unpaired t test and one- and two-way ANOVA with post hoc Sidak or Dunnett test.

A simple dietary protein supplementation was sufficient to prevent chronic G108-mediated weight loss in obese mice (Figure 3A). Specifically, the high protein (HP; 40 kcal%) diet prevented the marked reduction in fat mass caused by G108 (Figure 3B). The HP diet also blunted the reduction in lean mass and grip strength; however, this became less pronounced over the course of the study (Figures 3B, S2B, and S2C). Importantly, the induction of hepatic amino acid catabolic pathways by G108 was not prevented by the HP diet (Figure 3C), thus demonstrating that the dietary intervention was not downregulating hepatic GCGR signaling. We found that G108 reduced the plasma abundance of most amino acids and that the protein supplementation was sufficient to rescue the majority of these (Figure 3D). Consistent with changes in feed efficiency (Figure S2D), analysis in metabolic cages demonstrated that G108 increased EE on a standard protein (SP), but not HP, diet (Figure 3E). These data suggest that the effects of G108 on whole-body energy balance may be mediated by changes in plasma amino acid availability.

Our observations bear striking similarity to the EE response induced by dietary protein or amino acid restriction. In these models, the components of EE are multi-faceted with increased sympathetic drive, motor activity, and thermogenic gene expression all implicated.^22^^,^^24^^,^^25^^,^^28^^,^^34^^,^^35^^,^^36^ The hepatic amino acid response (AAR) pathway, an evolutionary conserved response to low amino acids,^37^ is also activated by dietary protein or amino acid restriction.^22^^,^^23^ Indeed, we observed changes in the hepatic AAR pathway and components of EE in response to G108 on the SP, but not HP, diet (Figures 3F, S2E, and S3). Our data therefore point to a multi-system energy-consuming response to G108 that is prevented by dietary protein supplementation and has features of amino acid restriction paradigms.

### G108-mediated EE is a response to reduced plasma amino acids

To further validate the interaction between G108-stimulated hypoaminoacidemia and EE, we first conducted experiments in an acute setting. A single injection of G108 at the dose used in our chronic studies (7.5 nmol/kg) caused a transient reduction in plasma amino acids associated with a trend toward increased EE (Figures 3G and 3H). However, a higher dose of G108 caused a sustained reduction in plasma amino acids, and this was associated with a significantly increased EE response (Figures 3I and 3J). Importantly, mice fed the HP diet were protected against G108-stimulated hypoaminoacidemia and EE at both doses (Figures 3H and 3J). These findings therefore demonstrate that even in an acute setting, G108-mediated hypoaminoacidemia is co-incident with an increase in EE.

We then sought to gain further mechanistic insight into how a high protein diet can prevent glucagon-mediated EE and weight loss. We first formulated a diet where the additional casein within the HP diet was replaced by individual total amino acids (SP + TAA diet: formula 1; Table S3). This diet eliminated any unexplained effect of additional casein that was independent of amino acid content. Supplementing the standard protein diet with total amino acids prevented G108-mediated weight loss independent of food intake (Figures 3K, 3L, and S4A). This diet also rescued plasma amino acids (Figure 3M), prevented lean mass loss (Figure 3L), and activation of the AAR pathway (Figure S4B). It also prevented G108-induced EE (Figure 3N) and normalized feed efficiency (Figure S4C). These findings therefore demonstrate the specific mechanistic underpinning of G108-mediated EE and weight loss by low plasma amino acids.

In previous studies of dietary protein restriction, both essential amino acids (EAAs) and non-EAAs (NEAAs) have been implicated in the EE effects observed.^22^^,^^23^ We therefore asked whether the individual EAA or NEAA components of the SP + TAA diet (respectively, SP + EAA and SP + NEAA diets, formulae 2 and 3; Table S3) were sufficient to prevent G108-mediated EE and weight loss. Unlike total amino acid dietary supplementation (Figure 3K), we found that the G108-mediated effects on weight loss and feed efficiency were not suppressed when the standard protein diet was supplemented with either EAAs or NEAAs (Figure S5). These data therefore suggest that preventing total hypoaminoacidemia is a requirement for blunting G108-mediated EE and weight loss. Furthermore, these findings demonstrate that multiple amino acids may be involved in the EE response observed, and this is also in keeping with the divergent mechanisms of EE in response to individually restricted amino acids.^28^^,^^38^

To further confirm that hypoaminoacidemia underpins enhanced whole-body EE in response to G108, we manufactured a customized low amino acid diet (LAA diet: formula 5; Table S3). In it, we reduced the content of each amino acid to the same degree as we observed in the plasma of G108-treated mice receiving a standard protein diet (Figure 3D). We found that this diet was sufficient to reduce individual plasma amino acids (Figure 3O) and increase EE (Figure 3P). This was associated with activation of the hepatic AAR pathway, reduced feed efficiency, and prevention of weight gain (Figure S6). Taken together, these studies have revealed that a reduction in plasma amino acids is both a pre-requisite and a mechanistic explanation for glucagon-mediated EE and weight loss in the sub-anorectic range (Figure 3Q).

### G108 improves metabolic parameters in obese mice independent of EE and weight loss

**Figure 4:**
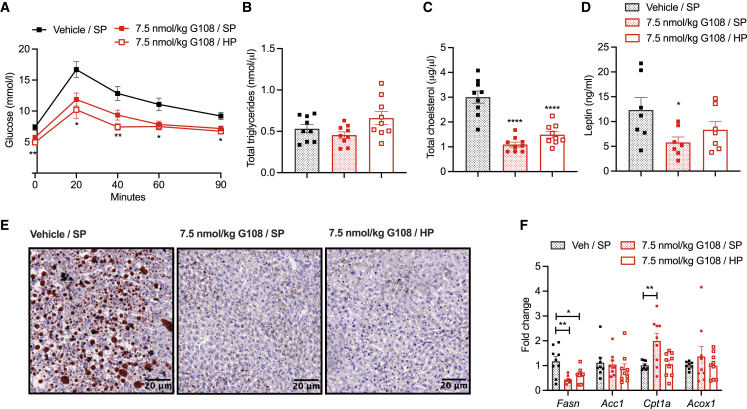
G108 improves metabolic parameters in obese mice independent of EE and weight loss Metabolic parameters from chronic 7.5 nmol/kg G108 daily dosing study in DIO mice as in Figure 3A. (A) Intraperitoneal glucose tolerance test (2 g/kg) at day 15 of study. n = 9/group. Significance indicates change compared with Veh/SP group. T = 0 min ∗∗p < 0.01 for 7.5 nmol/kg G108/HP, p = 0.062 for 7.5 nmol/kg G108/SP. T = 20 min ∗p < 0.05 all groups, T = 40 ∗∗p < 0.01 7.5 nmol/kg G108/HP, ∗p < 0.05 for 7.5 nmol/kg G108/SP. T = 60 and T = 90 min ∗p < 0.05 all groups. (B and C) Day 14 plasma (B) total triglyceride and (C) total cholesterol. n = 9/group. (D) Terminal plasma leptin on day 27 of study. n = 7/group. (E) Representative micrographs of terminal hepatic lipid content as indicated by oil red O staining. (F) Expression of markers of hepatic lipid metabolism, fatty acid synthase (*Fasn*)*,* acetyl-CoA carboxylase 1 (*Acc1*)*,* carnitine palmitoyltransferase (*Cpt1a*)*,* acyl-CoA oxidase 1 (*Acox1*). n = 8–9/group. ∗p < 0.05, ∗∗p < 0.01, ∗∗∗p < 0.001, ∗∗∗∗p < 0.0001. Data plotted as mean ± SEM, analyzed using one- and two-way ANOVA. Gene expression data analyzed by the 2^−ΔΔCT^ method and normalized to cyclophilin endogenous control.

We have found that a HP diet protects against lean mass loss but also prevents G108-mediated EE and total body weight loss (Figures 3A–3E). This may have clinical implications for use of glucagon as an obesity therapeutic. However, we found that weight loss is not required for G108 to improve several metabolic parameters. Indeed, chronic G108 treatment is remarkably efficient at improving glucose tolerance in obese mice, and this is independent of dietary protein content (Figure 4A). In keeping with a previous report on the effects of a glucagon analog,^39^ we also noted that following a transient increase, acute G108 administration caused a reduction in blood glucose that lasted several hours (Figures S8A and 1E). This effect was stable with chronic treatment and independent of dietary amino acid content (Figure S8B). Furthermore, despite no change in plasma triglycerides (Figure 4B), G108 treatment led to an improvement in plasma cholesterol (Figure 4C) on both SP and HP diets. Importantly, G108 was also highly effective at clearing liver fat in obese mice on both experimental diets (Figure 4E). The change in hepatic fat content in mice receiving G108 treatment was associated with changes in the expression of markers of hepatic lipid metabolism (Figure 4F), likely favoring fat oxidation and suppression of *de novo* lipogenesis consistent with the known effects of glucagon.^15^ These findings demonstrate that rescuing plasma amino acids, an intervention that prevents lean mass loss in G108-treated obese mice, does not blunt the beneficial effects of glucagon signaling on glycemia, plasma lipids, or hepatic steatosis.

Our data confirm that chronic glucagon treatment can increase EE and cause body weight loss independent of food intake. We provide compelling evidence that this is driven by a response to low plasma amino acid availability. It will be important to confirm whether these metabolic effects are recapitulated in humans in the context of obesity and type 2 diabetes, especially as multi-agonists targeting the GCGR are in clinical trials for metabolic disease indications.^40^ Recent reports have demonstrated a reduction in plasma amino acids in humans treated with these agonists.^26^^,^^41^^,^^27^ If clinical studies confirm that lean mass loss occurs with GCGR-targeted multi-agonists, simple strategies such as dietary protein supplementation may prevent this by rescuing hypoaminoacidemia. We also demonstrate that dietary protein supplementation will not prevent specific metabolic benefits of GCGR signaling, for example improved glycaemia and hepatic lipid metabolism. Lastly, our findings have implications for the development of multi-agonists for metabolic disease. We propose that a relatively small component of glucagon activity (supported by protein intake) can be used to drive metabolic improvements, while other components such as GLP1 and GIP activity can suppress appetite and contribute to improved glycaemia. It will be crucial that the relative balance of receptor activities is adjusted to obtain optimal metabolic effects.

### Limitations of the study

We have demonstrated that hypoaminoacidemia underpins the EE and body weight loss response to the glucagon analog G108. Although the signals mediating EE downstream to low plasma amino acid availability remain unclear, several multi-system pathways have been proposed.^28^^,^^38^ One of these involves activation of the hepatic AAR pathway leading to the induction of liver-derived FGF21.^22^^,^^36^^,^^42^ In our studies, using a relatively low dose of G108, a subtle induction of FGF21 is driven by hypoaminoacidemia. However, FGF21 is unlikely the sole contributor to the EE response to hypoaminoacidemia in our studies as there was no difference in EE between control and hepatic *Fgf21* knockout mice fed our rationally designed LAA diet (Figure S7). This is consistent with divergent mechanisms of hypoaminoacidemia-induced EE involving not only FGF21 but also sympathetic nervous system activation and central serotonergic mechanisms.^28^^,^^38^ Indeed, the partial FGF21-dependent effect of another glucagon analog on body weight is likely due to direct pharmacological stimulation of FGF21 transcription,^43^ which did not occur at the doses used in our studies. Therefore, future work may be required to elucidate the contribution of hypoaminoacidemia to EE and weight loss induced by GCGR-targeted agonists in development.

## STAR★Methods

### Key resources table

**Table:** 

REAGENT or RESOURCE	SOURCE	IDENTIFIER
**Chemicals, peptides, and recombinant proteins**		

Peptide G108	WuXi AppTec	N/A
Glucagon	Wuxi AppTec	N/A
GLP-1(7-36)NH_2_	Wuxi AppTec	N/A
Ethanol	Sigma-Aldrich	Cat# 8187601000
2-propanol	Sigma-Aldrich	Cat# I9516-1L
Lipofectamine 2000	Invitrogen	Cat# 11668-019
Tel Test Inc RNA STAT 60	Fisher Scientific	Cat# NC9256697
Oil Red O	Sigma-Aldrich	Cat# O0625-100G
HPLC grade water, Alfa Aesar, ultrapure	Fisher Scientific	Cat# 11338217

**Critical commercial assays**		

cAMP – GS dynamic kit	Cisbio	Cat# 62AM4PEC
Total amino acid assay kit	Sigma-Aldrich, Abcam	Cat# MAK002, ab65347
FGF21 ELISA kit	Abcam	Cat# ab229382
Triglyceride assay kit	Abcam	Cat# ab65336
Cholesterol assay kit	Abcam	Cat# ab65390
Leptin ELISA kit	Abcam	Cat# ab199082
Iscript cDNA synthesis kit	Bio-rad	Cat# 1708891
iTaq universal SYBRG Supermix	Bio-rad	Cat# 1725121

**Experimental models: Cell lines**		

HEK293T cells	European Collection of Authenticated Cell Cultures (ECACC)	RRID:CVCL_0063

**Oligonucleotides**		

GCG receptor cDNA ORF Clone, Mouse, untagged	Sinobiological	Cat# MG57952-UT
GLP-1 receptor cDNA ORF Clone, Mouse, untagged	Sinobiological	Cat# MG57391-UT
KiCqStart SYBR Green Primers	Merck	https://www.sigmaaldrich.com

**Experimental models: Organisms/strains**		

C57BL/6 mice	Charles River	Cat# C57BL/6NCrl
*Gcgr*^Δliver^ mice	Jackson Laboratories	N/A
*Fgf21*^Δliver^ mice	Jackson Laboratories	N/A
Software and algorithms		
SpectraMax i3x reader	Molecular Devices	
GraphPad Prism Version 9.0.0	GraphPad Software, Inc	https://www.graphpad.com/scientific-software/prism/
CalR Version 1.3	Banks lab 2021	https://calrapp.org
IBM SPSS Statistics Version 28	IBM	https://www.ibm.com/uk-en/analytics/spss-statistics-software

**Other**		

Custom Diets	Research Diets, Inc	Cat# D11112201, D08091803, D08091801, D12492, Formula 1–5 (Table S3)
Diets	Special Diet Services	Cat# RM1 diet
GlucoRx glucose monitor	GlucoRx	Cat# GlucoRx Nexus
Grip strength meter	Bioseb	Cat# Bio-GS3
MRI	EchoMRI	EchoMRI-100H
Metabolic cages	Columbus Instruments	Comprehensive Laboratory Animal Monitoring System (CLAMS)

### Resource availability

#### Lead contact

Further enquiries regarding resources and reagents should be directed to the lead contact, Tricia M.M. Tan (t.tan@imperial.ac.uk).

#### Material availability

This work did not generate new unique reagents.

### Experimental model and subject details

#### Animal studies

All animal studies were conducted according to the Animals (Scientific Procedures) Act 1986 Amendment Regulations 2012 and approved by the Animal Welfare Ethical Review Body at Imperial College London. For acute and chronic studies, lean 8-14-week-old male C57BL/6 mice and 8-week-old male Wistar Rats were purchased from Charles River, Margate, UK. Hepatic specific glucagon knockout mice (*Gcgr*^Δliver^) were obtained from Jackson Laboratories. *Gcgr*^*flox/flox*^ mice were crossed with hemizygous *Albumin-Cre* mice, generating *Gcgr*^*flox/flox*^*:Alb-Cre* + (*Gcgr*^Δliver^) and *Gcgr*^*flox/flox*^*:Alb-Cre-* (control) mice. Hepatic specific FGF21 knockout mice (*Fgf21*^Δliver^) were also obtained from Jackson Laboratories. *Fgf21*^*flox/flox*^ mice were crossed with hemizygous *Albumin-Cre* mice, generating *Fgf21*^*flox/flox*^*:Alb-Cre* + (*Fgf21*^Δliver^) and *Fgf21*^*flox/flox*^*:Alb-Cre-* (control) mice. *Cre* recombinase status of mice was confirmed by genotyping analysis. All animals had *ad lib* access to food/water and were individually housed in a temperature-controlled facility, under a standard 12-h light/dark cycle with lights on at 0730 am. Littermates were randomly assigned to experimental groups.

#### Cell lines

HEK293T cells were cultured in DMEM +10% FBS +1% penicillin/streptomycin at 37°C 5% CO2. Following cell preparation and seeding, HEK293T cells were stably transfected with mouse glucagon receptor (mGCGR) or GLP-1 receptor (mGLP-1), purchased from Sinobiological. Plasmids were initially reconstituted in nuclease-free water to a concentration of 200 ng/μL. Lipofectamine 2000 reagent (Invitrogen) was used to aid DNA encapsulation and cell transfection. Cells were incubated for 48 h prior to a cAMP assay. Following serial dilution of G108 and native ligands, cells were stimulated for 30 min at 37°C prior to measurement of cAMP accumulation assay using the cAMP-GS dynamic assay kit (Cisbio) and a SpectraMax i3x reader.

### Method details

#### Peptides

Peptide G108 is a custom peptide obtained from WuXi AppTec, China, based on the amino acid sequence of oxyntomodulin. By altering the amino acid sequence of native oxyntomodulin, the predicted potency at the glucagon receptor is enhanced, whilst reducing GLP-1 activity. A long-acting preparation of the peptide was achieved by the addition of a proline-linked N-terminal octadecanoic fatty acid. G108 was synthesised by using solid-phase peptide synthesis, with each amino acid sequentially added from the C to the N terminus. The peptide was purified using reverse phase preparative high performance liquid chromatography (HPLC) followed by lyophilisation. Purity was determined by reverse-phase HPLC and by matrix assisted laser desorption ionization mass spectroscopy (MALDI-MS). For all *in vivo* studies, G108 was diluted in HPLC grade water prior to subcutaneous administration. Glucagon and GLP-1 were also purchased from Wuxi AppTec.

#### Diets

For acute studies, a standard RM1 diet (Special Diet Services, UK) was used. For chronic studies and single dosing studies in metabolic cages, custom diets were obtained (Research Diets, Inc) and are detailed in Tables S2 and S3. For the chronic studies in lean mice, OpenStandard Diet D11112201 was used as a ‘Standard protein’ diet, consisting of 20 kcal% protein. For the chronic study in diet-induced obese (DIO) mice, OpenStandard Diet D08091803 was used as a high fat/standard protein (SP) diet consisting of 45 kcal% fat and 20 kcal% protein (Table S2). The isocaloric high fat/high protein diet, D08091801 was used as the high protein (HP) intervention diet, consisting of 45 kcal% fat and 40 kcal% protein (Table S2). A standard DIO series diet (D12492) consisting of 60 kcal% fat and 20 kcal% protein was used in chronic DIO studies to develop DIO status in C57BL/6 mice (Table S2). For the chronic mechanistic studies, custom diets with varying amino acid amounts were used (Research Diets, Inc.: Table S3).

#### G108 induced acute metabolism of glucose, amino acids and urea cycle enzyme expression

Twelve weight-matched 12-week-old lean C57BL/6 mice were fasted for 2 h prior to a single intraperitoneal injection of 10 nmol/kg dose of G108 diluted in sterile HPLC water (pH > 7), or vehicle control alone. Tail venesection was performed at baseline, 15, 30, 60 and 120 min and blood glucose measured using a handheld GlucoRx meter. For hepatic carbohydrate and amino acid metabolism gene expression analysis, weight-matched 12-week-old lean C57BL/6 mice were subcutaneously administered with 20 nmol/kg G108 diluted in sterile HPLC water (pH > 7), or vehicle control via subcutaneous injection at 9 am (N = 8). All animals had *ad libitum* access to RM1 diet and water. Liver tissue was dissected at 24 h and stored at −80°C prior to hepatic gene expression analysis.

#### Pharmacokinetic profile of peptide G108

10-week-old male Wistar rats (N = 4) were administered with 0.5 mg G108 via 100 μL subcutaneous injection. Tail venesection was performed to collect 100 μL blood samples prior to injection and at 1.5, 3, 6, 24, 48 and 72 h after injection. Blood samples were immediately frozen on ice, centrifuged at 8000 × g for 10 min at 4°C, and the plasma supernatant collected. Samples were stored at −20°C.

An in-house radioimmunoassay was used to determine peptide concentrations in the rat plasma. Antiserum (RA553/3) was produced in rabbits against the purified glucagon (1–16) fragment which specifically cross-reacted with the N-terminal glucagon-like sequence of G108. Direct iodination was used to produce Iodine-125 glucagon which was purified by reverse phase high pressure liquid chromatography. The assay was performed in a total volume of 700 μL of 0.06 M phosphate EDTA buffer containing 0.3% BSA. All samples were assayed in duplicate and the standard curve was set using 5 pmol/mL G108 in assay buffer. The assay was incubated for 3 nights at 4°C prior to separation via charcoal into free and antibody-bound label. A γ-counter was used to count the pellet and supernatant and the ratio of free to bound label was calculated. The plasma peptide concentration was calculated using two phase exponential decay with respect to the relevant standard curve.

#### Dose-finding and chronic low-dose G108 study in lean mice

C57BL/6 mice matched for weight and age (14-week-old, N = 24) were administered via subcutaneous injection at 9 am daily for 18 days, vehicle control or G108 diluted in sterile HPLC water. Ascending doses of either 5 nmol/kg, 10 nmol/kg or 20 nmol/kg G018 were administered. All mice had *ad libitum* access to RM1 diet (Special Diet Services, UK) and water. Food intake and body weights were recorded daily.

For the low-dose G108 (5 nmol/kg) study, C57BL/6 mice matched for weight and age (14-week-old, N = 14) were single housed and split into two treatment groups (N = 7/group). All mice were subcutaneously administered with the allocated treatment daily at 9 am for 21 days. Mice were administered with vehicle control (HPLC water) or 5 nmol/kg G108 (diluted in sterile HPLC water). All mice had *ad libitum* access to a standard protein diet containing 20 kcal% protein (diet D11112201) (Table S2). Food intake and body weight were measured daily prior to treatment each day. EchoMRI analysis was carried out at baseline and days 7, 14 and 21. Tail venesection was carried out on day 7 of study, pre- and 4 h post treatment dose. Blood samples were immediately transferred on ice, centrifuged at 2800 × g for 10 min prior to plasma total amino acid analysis. Forelimb grip strength testing was performed at baseline, day 14 and day 21. On day 21, following a 4 h fast, mice were culled, and the following muscle groups dissected and weighed: quadriceps, gastrocnemius, soleus, tibialis anterior, extensor digitorum longus.

#### Hepatic-specific glucagon receptor knockout mouse chronic study

Age-matched (12-week-old) *Gcgr*^*flox/flox*^*:Alb-Cre* + (*Gcgr*^Δliver^) and *Gcgr*^*flox/flox*^*:Alb-Cre-* (control) mice were subcutaneously administered daily with either 5 nmol/kg G108 or vehicle control (HPLC water) for 14 days (N = 7–8/group). All mice were single-housed and had *ad libitum* access to water and a standard (20 kcal%) protein diet (diet D11112201, Table S2). Food intake and body weight were measured daily prior to administration of peptide each day. EchoMRI analysis was carried out at baseline and day 14. Tail venesection was carried out 4-h post-dose on day 14. Blood samples were immediately transferred on ice, centrifuged at 2800 × g for 10 min prior to plasma total amino acid analysis.

#### Chronic low-dose G108 studies in DIO mice

Lean 10-week-old C57BL/6 mice (N = 27) were provided *ad libitum* a 60 kcal% high fat diet (diet D12492, Table S2) for 12 weeks prior to the start of the study. The cohort of mice were individually housed and divided into three treatment groups, based on treatment and diet allocation: Vehicle/High fat, Standard protein diet (SP) (N = 9), 7.5 nmol/kg G108/SP diet (N = 9) and 7.5 nmol/kg G108/High fat, High protein diet (HP) (N = 9). The high fat, standard protein diet (SP diet) (D08091803) and high fat, high protein diet (HP diet) (D08091801) are detailed in Table S2. All mice had *ad libitum* access to water and their allocated diets. Mice were subcutaneously administered with their respective treatments at 9 am each day for 27 days. Food intake, water intake and body weight were measured using the same weighing scale each morning prior to vehicle or G108 administration. EchoMRI was carried out at baseline, day 14 and day 25. Tail venesection was carried out on day 13 of the study immediately pre-treatment. 40–50 μL of blood was collected and immediately transferred on ice, centrifuged at 2800 × g for 10 min prior to analysis for total and individual plasma amino acids. A glucose tolerance test was carried out on day 15 of the study following a 5 h fast as described below. Forelimb grip strength testing was performed at baseline and day 25 of the study. After 20 days, all groups were placed into metabolic cages, single housed for 72 h, as described below. After 72 h, all groups were removed from the metabolic cages and returned to their home cages. Following a 4 h fast on day 27 all mice were culled, with the following tissues dissected and snap frozen in liquid nitrogen prior to storage at −80°C: gastrocnemius, quadriceps, brown adipose tissue (BAT) and liver. Terminal blood was also collected and transferred on ice immediately for centrifugation as previously described and storage at −80°C.

#### Single dose G108 EE studies in lean mice

Lean 10-week-old C57BL/6 mice (N = 20) were provided *ad libitum* a 60 kcal% high fat diet (diet D12492, Table S2) for 10 days. They were then switched to either a standard protein high fat diet (D08091803) or a high protein high fat diet (D08091801) (N = 10/group) for 1 week prior to commencing the study. All mice were then placed into metabolic cages, individually housed, and allowed to acclimatise for 24 h. On day 2, baseline metabolic readings were obtained prior to single dose administration of 7.5 nmol/kg G108 on day 3 at 9 am. Metabolic readings were subsequently obtained over the following 48 h prior to single dose administration of 20 nmol/kg G108 on day 5. On day 8, all mice were removed from the metabolic cages and returned to their home cages. A parallel study was then carried out whereby venous blood samples were collected at baseline and daily following single dosing with either 7.5 nmol/kg or 20 nmol/kg G018. Blood samples were transferred on ice immediately for centrifugation as previously described. Samples were stored at −80°C prior to measurement of plasma total amino acids.

#### Total amino acid supplementation in G108-treated DIO mice

Lean 10-week-old C57BL/6 mice (N = 32) were provided *ad libitum* a 60 kcal% high fat diet (diet D12492, Table S2) *ad libitum* for 8 weeks. Half of the animals were then provided with a high fat standard protein diet (D08091803, Table S2) or a high fat standard protein diet supplemented with additional total amino acids (SP + TAA diet, Formula 1, Table S3). From each diet group, animals were allocated to receive either 7.5 nmol/kg G108 or vehicle control (N = 8/group). The subsequent four treatment groups were as follows: Vehicle/SP diet, G108/SP diet, Vehicle/SP + TAA diet, G108/SP + TAA diet. Food intake and body weight was recorded every 48 h during the study. Baseline body composition analysis (EchoMRI) was carried out in addition body composition analysis on day 18. Tail venesection was carried out on day 16 of the study 4 h following respective treatments. 40–50 μL of blood was collected and immediately transferred on ice, centrifuged at 2800 × g for 10 min prior to measurement of plasma total amino acids. A glucose time-course profile was carried out on day 0 and day 19 after administration of respective treatments. All animals were placed into metabolic cages for 72 h on day 20 for assessment of EE.

#### Essential and non-essential amino acid supplementation in G108-treated DIO mice

Lean 10-week-old C57BL/6 mice (N = 48) were provided *ad libitum* with a 60 kcal% high fat diet (diet D12492, Table S2) for 8 weeks. They were then provided with either a high fat standard protein diet (SP) (D08091803), a high fat standard protein diet supplemented with essential amino acids (SP + EAA) (Formula 2, Table S3), or a high fat standard protein diet supplemented with non-essential amino acids (SP + NEAA) (Formula 3, Table S3), N = 16/group. Mice from each diet group received either 7.5 nmol/kg G108 or vehicle control resulting in the following six treatment groups, N = 8/group: Vehicle/SP, 7.5 nmol/kg G108/SP, Vehicle/SP +EAA, 7.5 nmol/kg G108/SP + EAA, Vehicle/SP + NEAA, 7.5 nmol/kg G108 + NEAA. Food intake and body weight was measured every 48 h. Prior to commencing their respective diets, baseline body composition analysis was (EchoMRI) was carried out in addition to body composition analysis on day 11. Tail venesection was carried out on day 13 of the study 4 h following respective treatments. 40–50 μL of blood was collected and immediately transferred on ice, centrifuged at 2800 × g for 10 min, prior to measuring plasma FGF21.

#### Dietary restriction of amino acids matched to G108-treated DIO mice

Lean 10-week-old C57BL/6 mice (N = 15) were provided *ad libitum* with a 60 kcal% high fat diet (diet D12492, Table S2) for 8 weeks. They were then provided with either an amino acid-based standard protein high fat diet (N = 7) (SPAA diet: Formula 4, Table S3) or a low amino acid high fat diet (N = 8) (LAA diet: Formula 5, Table S3). The low amino acid diet (Formula 5) was designed to match the reduction in plasma individual amino acids caused by chronic G108 (7.5 nmol/kg) administration in DIO mice (as in Figure 3D). Specifically, the percentage reduction of each plasma amino acid caused by chronic G108 administration was applied to individual amino acid (g%) of the SPAA diet, to create the LAA diet. Prior to switching to these diets, baseline body composition analysis (EchoMRI) was carried out in addition to body composition analysis on day 14 of commencing the respective diets. On day 26, all animals were placed into metabolic cages for assessment of EE over a 24-h period. Terminal blood collection was carried out on day 34 of the study for measurement of plasma individual amino acid and FGF21.

#### Dietary restriction of amino acids in hepatic specific *Fgf21* knockout mice

Age-matched (10-week-old) lean *Fgf21r*^*flox/flox*^*:Alb-Cre* + (*Fgf21*^Δliver^) and *Gcgr*^*flox/flox*^*:Alb-Cre-* (control) mice were provided with a low amino acid (LAA) diet (Formula 5, Table S3) for two weeks (N = 7–8/group). On day 14, a tail venesection was carried out and plasma FGF21 was measured. Mice were then placed into metabolic cages for assessment of EE over a 48-h period.

#### Food intake, body weight and body composition

Food intake was measured daily using a dedicated weighing scale and topped up accordingly. Body weight was recorded at 9 am daily or every 48 h using the same weighing scale. Body composition was measured using an EchoMRI analyser at various timepoints during chronic studies. Body composition measurements were taken on the same time of day to reduce intra-animal variability. Animals were placed in a transparent cylindrical Perspex tube and both ends secured with Perspex clips. The tubes containing the animal was then carefully placed into the MRI machine (EchoMRI −100H analyser, EchoMRI). Each scan lasted approximately 90 s where lean mass and fat mass were recorded in grams. Following the scan, the mice were removed from the Perspex tube and returned to their respective cages.

#### Glucose tolerance tests

For glucose tolerance tests, following a 5 h fast and administration of peptide or vehicle treatment, mice were administered with glucose by intraperitoneal injection at a dose of 2 g/kg body weight. Tail vein blood samples were taken at baseline, 20, 40, 60 and 90 min and glucose measured (mmol/L) using a handheld GlucoRx monitor (GlucoRx, UK).

#### Muscle function analysis – grip strength

A grip strength meter (Bioseb, France) was used to measure grip strength. Mice were individually lowered on to a grip strength bar prior to gentle retraction of the tail until grip release. The maximum force, measured in g was recorded. Four consecutive measurements were made with a 1 min rest interval. An average of measurements was taken for each timepoint prior to subsequent analysis.

#### Energy expenditure studies

To measure energy expenditure, DIO mice were placed into a Comprehensive Laboratory Animal Monitoring System (CLAMS, Columbus instruments, Columbus, US). Measurement of oxygen consumption and carbon dioxide production was taken at 20 min intervals. Food intake was also measured electronically without the need to remove food. Each cohort of mice were placed into the CLAMS system with daily weighing of body weight prior to administration of the allocated treatment at 9 am. Following administration of treatment, mice were placed back into the metabolic cages with *ad libitum* access to their allocated diet. For studies with the low amino acid (LAA) diet in metabolic cages, animals were not removed from the metabolic cages until the end of the experiment and had *ad libitum* access to either standard protein or low amino acid diets. Feed efficiency was calculated as mg body weight change per kJ consumed.

#### Biochemical analysis

Plasma total amino acids were measured using fluorometric-based ELISA kits as described (MAK002, Sigma-Aldrich, Ab65347, Abcam). Plasma FGF21 was measured using a fluorescent based ELISA kit (ab229382, Abcam). Plasma triglyceride, cholesterol and leptin were measured using ELISA kits (ab65336, ab65390, ab199082, Abcam). Individual amino acids were measured using Gas Chromatography/Mass Spectrometry (WellChild laboratory, Evelina Children’s Hospital, St Thomas’s Hospital, London).

#### Liver histology for lipid analysis

Frozen liver samples were sectioned and at 8–10 mm and fixed in formalin. Sections were stained with Oil Red O according to standard protocols, prior to imaging. Representative samples are shown.

#### Targeted quantitative polymerase chain reaction analysis

RNA was extracted from liver, muscle and BAT tissue using 1 mL TRIzol added to samples prior to homogenisation within a mechanical homogeniser for 2–3 min. Chloroform was added to each individual sample prior to gentle shaking and centrifugation at 16000 × g for 15 min. The supernatant was decanted into separate Eppendorf tubes and a 1:1 mixture of supernatant to 70% iso-propanol was made prior to further centrifugation at 16000 × g for 15 min. Following this the RNA pellet was washed three times with 70% ethanol and resuspended in sterile RNase-free water. RNA was quantified in addition to quality assessed using a NanoDrop spectrophotometer and 1 μg of RNA was then used for the synthesis of cDNA using the iScript cDNA synthesis Kit (Bio-Rad). Quantitative polymerase chain reaction (qPCR) was then undertaken using the SYBR-Green method using KiCqStart SYBR Green primers (Merck).

### Quantification and statistical analysis

All statistical analysis was conducted in GraphPad Prism 9.0.0 (GraphPad Software) or IBM SPSS Statistics (IBM Corp. Released 2021. IBM SPSS Statistics for Macintosh, Version 28.0. Armonk, NY: IBM Corp). Statistical significance levels and n numbers are reported in figure legends in addition to dispersion represented as mean and SEM. Significance is defined as p values below 0.05. Assumptions for parametric tests included normal distribution of datasets. Analysis of metabolic variables was conducted using CalR Version 1.3^44^ and SPSS in accordance with published guidelines^45^ using regression based ANCOVA with lean or total mass as a covariate. EE data plotted as predicted values with mean ± SEM, significance denotes result of ANCOVA analysis.

## Data Availability

• Data reported in this paper will be shared by the lead contact upon request. • This paper does not report original code. • Any additional information required to reanalyse the data reported is available from the lead contact upon request.

## Supplemental information

Document S1. Figures S1–S8 and Tables S1–S3

Document S2. Article plus supplemental information
