# Supplementary material for: Hypoaminoacidemia underpins glucagon-mediated energy expenditure and weight loss
Source: Cell Rep Med. 2022 Nov 15;3(11):100810. doi: 10.1016/j.xcrm.2022.100810 (PMC9729826; doi:10.1016/j.xcrm.2022.100810)
Supplement: Document S1. Figures S1–S8 and Tables S1–S3 [file mmc1.pdf]

Cell Reports Medicine, Volume *3*

Supplemental information

Hypoaminoacidemia underpins glucagon-mediated energy expenditure and weight loss

David C.D. Hope, Charlotte E. Hinds, Tatiana Lopes, Matthew L. Vincent, Jed V. Shrewsbury, Arthur T.C. Yu, Iona Davies, Rebecca Scott, Ben Jones, Kevin G. Murphy, James S. Minnion, Alessandro Sardini, David Carling, Thomas A. Lutz, Stephen R. Bloom, Tricia M.M. Tan, and Bryn M. Owen

Supplemental data

	G108	GCG	GLP-1
mGCGR	10.1 ± 0.1	9.5 ± 0.5	n.d.
mGLP-1R	8.2 ± 0.3	n.d.	9.7 ± 0.1

Table S1: GLP-1 and Glucagon receptor potencies for G108. Related to Figure 1. Murine GLP-1R and GCGR cAMP accumulation assay with pEC_{50} ($-\log_{10}$ of EC50 in M) for G108 and endogenous ligands glucagon (GCG) and GLP-1(7-36)NH_2, 4 replicates per ligand or G108. n.d. = not determined.

**Diet**	**Carbohydrate (kcal%)**	**Fat (kcal%)**	**Protein (kcal%)**
**D12492**	20	60	20
**D11112201**	65	15	20
**D08091803 (SP diet)**	35	45	20
**D08091801 (HP diet)**	15	45	40

Table S2: Macronutrient composition of casein-based diets. Related to Figures 2, 3 and 4. Composition of custom diets (Research Diets, Inc) used for chronic studies in lean and DIO mice. High and low protein diets identical in caloric density. Values represent kcal% composition.

Constituent	**Formula 1**		**Formula 2**		**Formula 3**		**Formula 4**		**Formula 5**	
	200 g casein + total amino acids from 200 g casein (SP+TAA diet)		200 g casein + EAA from 200 g casein (SP+EAA diet)		200 g casein + NEAA from 200 g casein (SP+NEAA diet)		Amino acid version of D08091803. (SPAA diet)		GCMS matched low amino acid diet (LAA diet)	
	g%	kcal%	g%	kcal%	g%	kcal%	g%	kcal%	g%	kcal%
Protein	44	37	32	27	35	30	20	17	11	10
Carbohydrate	21	18	33	28	29	25	44	38	53	45
Fat	23	45	23	45	23	45	23	45	23	45
Kcal/g		4.69		4.69		4.69		4.69		4.69
Casein	200	800	200	800	200	800	0	0	0	0
L-Histidine	4.5	18	4.5	18	0	0	4.5	18	3.3	13
L-Isoleucine	7.5	30	7.5	30	0	0	7.5	30	4.5	18
L-Leucine	15.7	63	15.7	63	0	0	15.7	63	9.7	39
L-Lysine	13	52	13	52	0	0	13	52	3.4	13
L-Methionine	5	20	5	20	0	0	5	20	1.5	6
L-Phenylalanine	8.3	33	8.3	33	0	0	8.3	33	6.5	26
L-Threonine	7.1	28	7.1	28	0	0	7.1	28	1.8	7
L-Tryptophan	2.1	8	2.1	8	0	0	2.1	8	1.6	6
L-Valine	9.2	37	9.2	37	0	0	9.2	37	5.3	21
L-Alanine	5	20	0	0	5	20	5	20	2	8
L-Arginine	5.9	24	0	0	5.9	24	5.9	24	2.8	11
L-Asparagine	7	28	0	0	7	28	7	28	2.1	8
L-Aspartic acid	5	20	0	0	5	30	5	20	4.5	18
L-Cystine	4.2	17	3	12	4.2	17	4.2	17	4.2	17
L-Glutamine	17	68	0	0	17	68	17	68	9.1	37
L-Glutamic acid	20.7	83	0	0	20.7	83	20.7	83	17.6	70
Glycine	3	12	0	0	3	12	3	12	1.4	6
L-Proline	17.6	70	0	0	17.6	70	27.6	70	8.1	33
L-Serine	9.9	40	0	0	9.9	40	9.9	40	4	16
L-Tyrosine	9	36	0	0	9	36	9	36	4.6	19
Corn Starch	3.1	12	104.4	418	75.5	302	201.3	805	279.9	1120
Maltodextrin 10	100	400	100	400	100	400	100	400	100	400

Sucrose	68.8	275	68.8	275	68.8	275	68.8	275	68.8	275
Cellulose	50	0	50	0	50	0	50	0	50	0
Soybean Oil	25	225	25	225	25	225	25	225	25	225
Lard	177.5	1598	177.5	2598	177.5	1598	177.5	1598	177.5	1598
Mineral Mix	10	0	10	0	10	0	10	0	10	0
DiCalcium Phosphate	13	0	13	0	13	0	13	0	13	0
Calcium Carbonate	5.5	0	5.5	0	5.5	0	5.5	0	5.5	0
Potassium Citrate, 1 H20	16.5	0	16.5	0	16.5	0	16.5	0	16.5	0
Sodium Bicarbonate	7.5	0	7.5	0	7.5	0	7.5	0	7.5	0
Vitamin Mix	10	40	10	40	10	40	10	40	10	40
Choline Bitrartrate	2	0	2	0	2	0	2	0	2	0
Yellow dye	0	0	0.05	0	0	0	0.025	0	0	0
Red dye	0.025	0	0	0	0.05	0	0	0	0	0
Blue dye	0.025	0	0	0	0	0	0.025	0	0.05	0
Total	865.7	4057	865.7	4057	865.7	4057	863.9	4050	863.9	4050

Table S3: Composition of amino acid-based diets. Research Diets, Inc. Related to Figure 3.

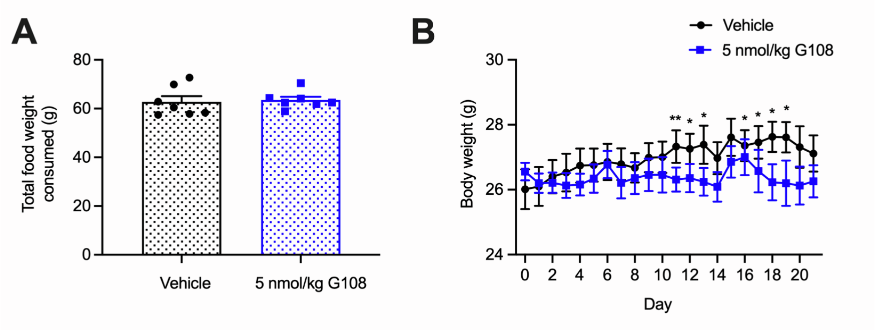

Figure S1: G108 causes suppression of weight gain in lean mice. Related to Figure 2. Body weight over 21 days with daily dosing of 5 nmol/kg G108 or vehicle control in lean C57BL/6 mice n=7/group. **(A)** Total food weight consumed (g). **(B)** Body weight, significance indicates change from baseline. *$p<0.05$, data expressed as mean ± SEM. Analysed using t-test and two-way ANOVA with post hoc Sidak's test.

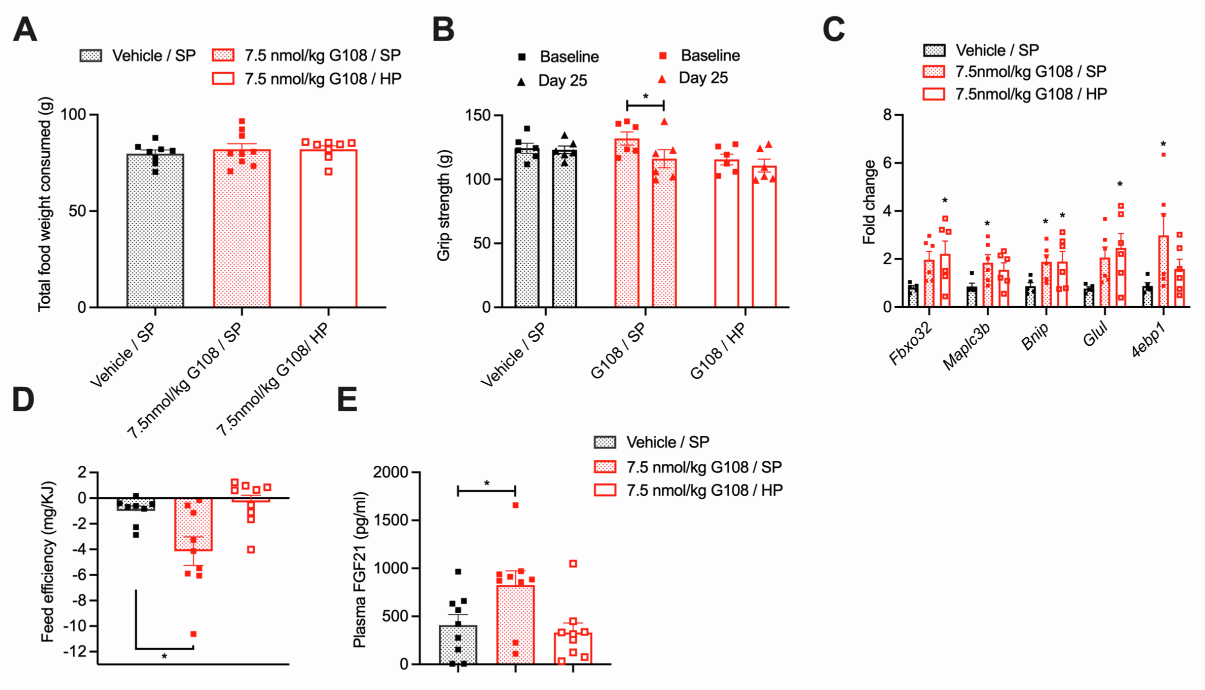

Figure S2: Dietary protein supplementation prevents G108-mediated functional muscle loss and activation of the hepatic AAR, through rescue of hypoaminoacidemia in obese mice. Related to Figure 3. **(A)** Total food weight consumed in DIO mice over 27 days, n=8-9/group. **(B)** Forelimb grip strength on day 25 of study, n=6/group. **(C)** Expression of markers of proteolysis in quadriceps muscle, significance indicates change from vehicle group, n=5-6/group. **(D)** Feed efficiency over 20 days of study, n=8-9/group. **(E)** Terminal plasma FGF21, n=9/group. SP–standard protein diet, HP–high protein diet (Table S2). *p<0.05, **p<0.01. Data expressed as mean ± SEM, analysed using one- and two-way ANOVA with post hoc Sidak and Dunnett tests. Gene expression data analysed by the $2^{-\Delta\Delta CT}$ method, normalised to beta-actin endogenous control.

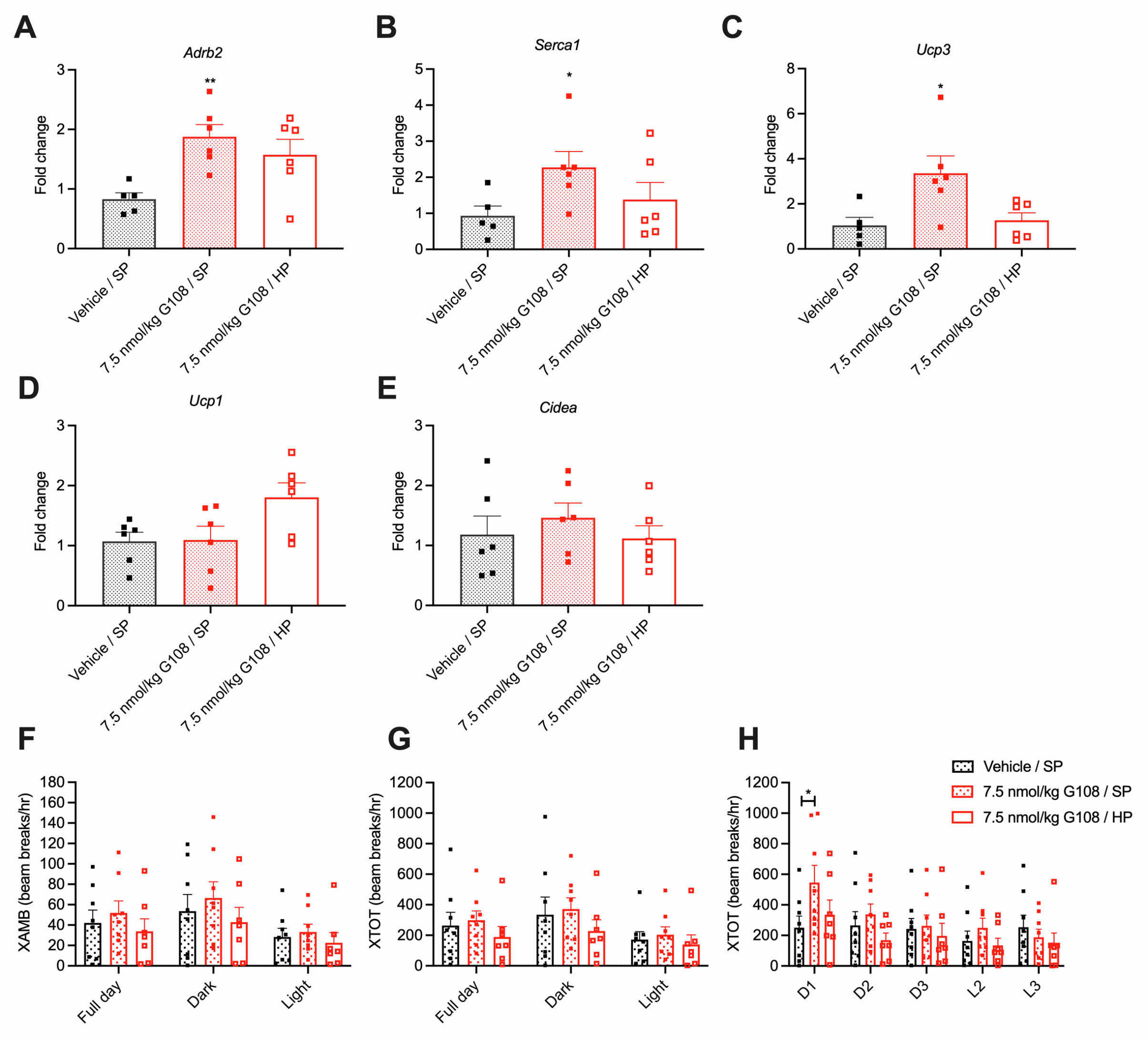

Figure S3: Dietary protein supplementation prevents upregulation of EE mechanisms in response to G108 treatment. Related to Figure 3. Markers of increased EE from 27-day daily dosing study with G108 in diet induced obese (DIO) mice**. (A-C)** Gene expression in gastrocnemius muscle, N=6/group. **(A)** Beta 2 adrenergic receptor (*Adrb2*) **(B)** Sarcoplasmic/endoplasmic reticulum calcium ATPase 1 (*Serca1*) **(C)** Uncoupling protein 3 (*Ucp3*) **(D-E)** Gene expression changes in brown adipose tissue (BAT), N=6/group. **(D)** Uncoupling protein 1 (*Ucp1*) **(E)** Cell Death Inducing DFFA Like Effector A (*Cidea*). **(F)** Ambulatory activity (XAMB) over 60 hours in 7.5 nmol/kg G108-treated mice. **(G)** Locomotor activity (XTOT) over 60 hours in 7.5 nmol/kg G108 treated mice. **(H)** Locomotor activity over individual dark and light periods in 7.5 nmol/kg G108-treated mice, n=7-8/group. SP–standard protein diet, HP–high protein diet (Table S2). *p<0.05, **p<0.01. Data expressed as mean ± SEM analysed using one-way ANOVA with post hoc Sidak's test, difference between treatment and vehicle group. Gene expression data analysed by the $2^{-\Delta\Delta CT}$ method, normalised to beta-actin endogenous control.

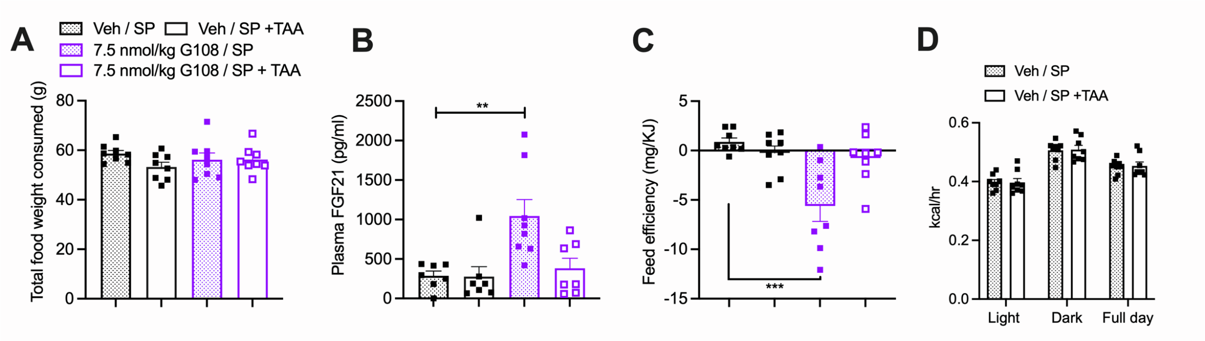

Figure S4: Total amino acid dietary supplementation does not alter food intake and supresses the effects of G108 on FGF21 and feed efficiency in obese mice. Related to Figure 3. SP-standard protein diet (Table S2), SP+TAA-standard protein diet with additional total amino acids (Formula 1, Table S3), n=8/group. **(A)** Total food intake at day 20 of study. **(B)** Plasma FGF21 on day 16 of study **(C)** Feed efficiency over 20 days **(D)** Average energy expenditure (kcal/hr) between Veh / SP and Veh / SP+TAA groups. *p<0.05, *p<0.01, ***p<0.001. Data plotted as mean ± SEM, analysed using one-way ANOVA with post hoc Dunnett's test.

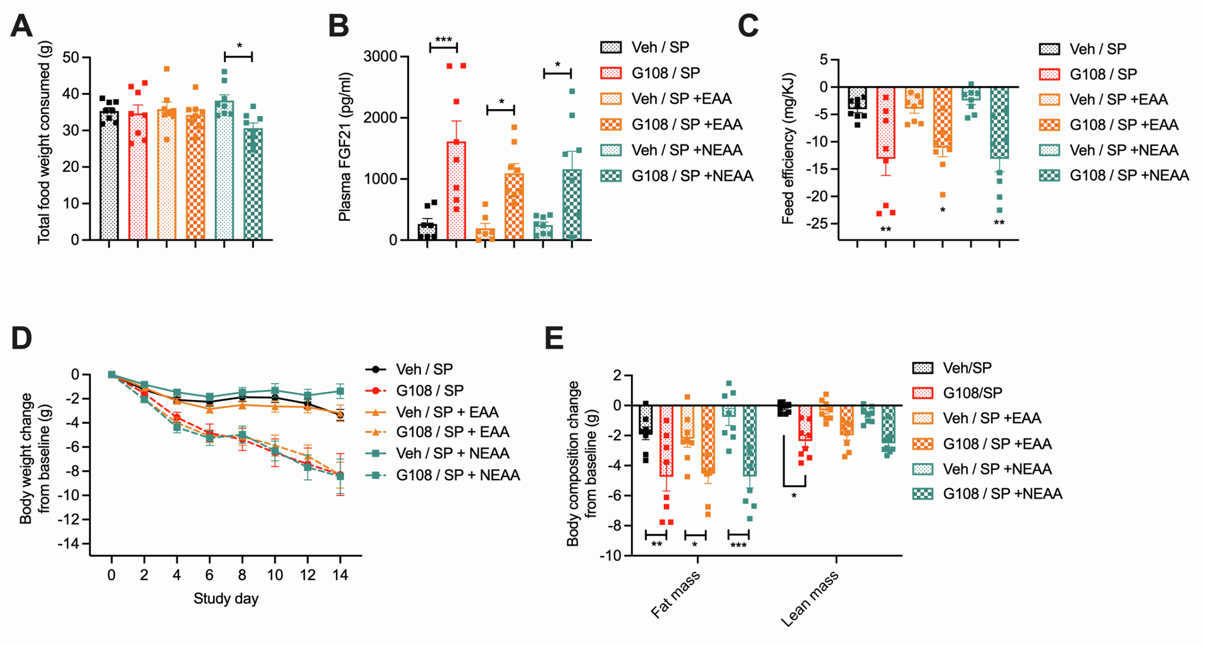

Figure S5: Essential or non-essential amino acid dietary supplementation does not supress the effect of G108 on FGF21, feed efficiency, body weight or body composition in obese mice. Related to Figure 3. Daily administration for 14 days with either vehicle control or 7.5 nmol/kg G108. SP-standard protein diet, SP+EAA-standard protein diet with additional essential amino acids, SP+NEAA-standard protein diet with additional non-essential amino acids (Formulae 2 and 3 respectively, Table S3), n=8/group. **(A)** Total food intake at day 14 of study. **(B)** Plasma FGF21 on day 13 of study. **(C)** Feed efficiency over 14 days. **(D)** Body weight change over 14 days from baseline. **(E)** Change in body composition at day 11 compared to baseline. *p<0.05, **p<0.01, ***p<0.001. Data plotted as mean ± SEM, analysed using one- and two-way ANOVA with post hoc Sidak and Dunnett's tests.

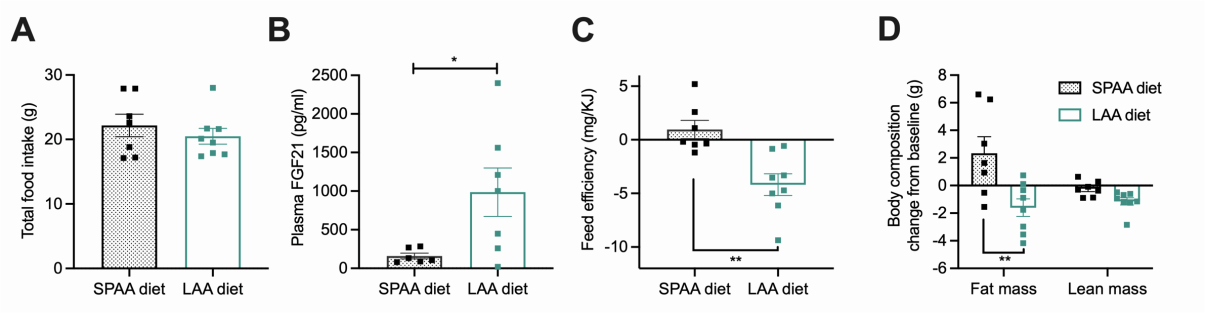

Figure S6: Effects of a low amino acid (LAA) diet on plasma FGF21, feed efficiency and body composition in obese mice. Related to Figure 3. SPAA-amino acid-based standard protein diet, LAA diet based on reduction of individual plasma amino acids in mice chronically treated with G108 (Figure 3D, Formulae 4 and 5 respectively, Table S3), n=7-8/group. **(A)** Total food intake over 7 days. **(B)** Terminal plasma FGF21. **(C)** Feed efficiency over 10 days. **(D)** Change in body composition at day 14 compared to baseline. *p<0.05, **p<0.01. Data plotted as mean ± SEM, analysed using unpaired t-test.

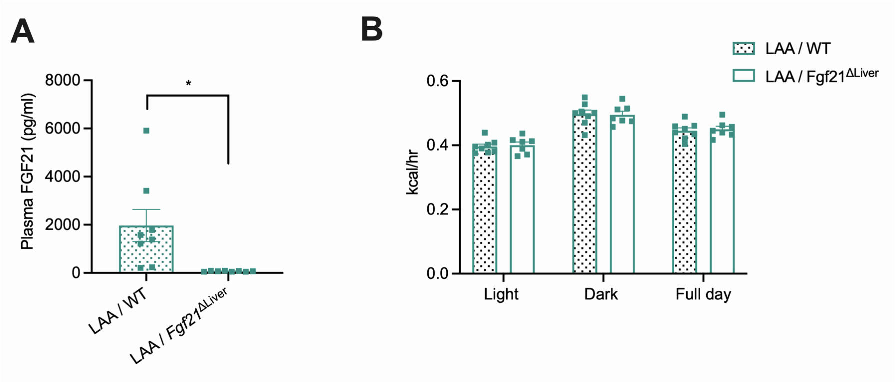

Figure S7: FGF21 and EE response in wild-type and *Fgf21*$^{\Delta liver}$ mice provided with a low amino acid (LAA) diet. Related to STAR Methods – Dietary restriction of amino acids in hepatic specific *Fgf21* knockout mice. LAA diet based on reduction of individual plasma amino acids in mice chronically treated with G108 (Formula 5, Table S3), n=7-8/group. **(A)** Plasma FGF21 on day 14. **(B)** Average energy expenditure (kcal/hr) over 48 hours, after 14 days of LAA diet. *p<0.05. Data plotted as mean ± SEM, analysed using unpaired t-test. EE data analysed with ANCOVA using lean mass as a covariate.

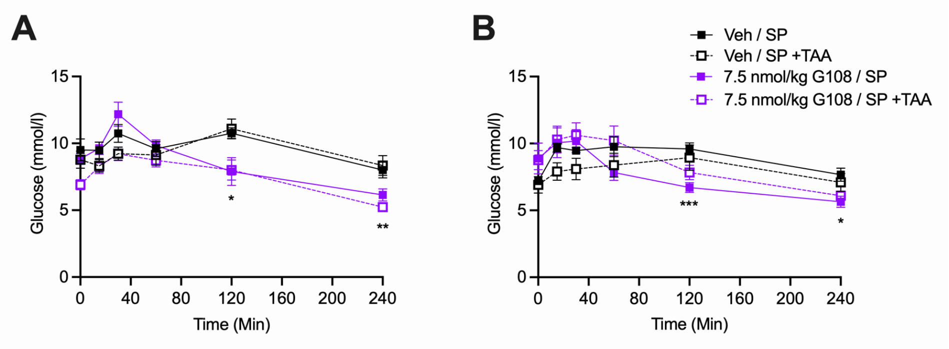

Figure S8: Glucose profiles following acute and chronic dosing of G108 in obese mice. Related to Figure 3. **(A)** Glucose profile over 4 hours following a single dose of 7.5 nmol/kg G018, n=8/group. **(B)** Glucose profile over 4 hours following a single dose of G018 after 19 days of daily dosing with 7.5 nmol/kg G108. *p<0.05, **p<0.01, ***p<0.001, compared to Veh / SP group. Data plotted as mean ± SEM, analysed using two-way ANOVA with post hoc Dunnett's test.
